# Arginine off-kilter: guanidinium is not as planar as restraints denote

**DOI:** 10.1107/S2059798320013534

**Published:** 2020-11-24

**Authors:** Nigel W. Moriarty, Dorothee Liebschner, Dale E. Tronrud, Paul D. Adams

**Affiliations:** a Molecular Biosciences and Integrated Bioimaging, Lawrence Berkeley National Laboratory, Berkeley, CA 94720, USA; b Department of Biochemistry and Biophysics and the Department of Chemistry, Oregon State University, Corvallis, OR 97331, USA; c Department of Bioengineering, University of California, Berkeley, CA 94720, USA

**Keywords:** arginine, chemical restraints, macromolecular refinement, guanidine, planarity

## Abstract

Crystallographic refinement of macromolecular structures relies on stereochemical restraints to mitigate the typically poor data-to-parameter ratio. For proteins, each amino acid has a unique set of geometry restraints which represent stereochemical information such as bond lengths, valence angles, torsion angles, dihedrals and planes. It has been shown that the geometry in refined structures can differ significantly from that present in libraries; for example, it was recently reported that the guanidinium moiety in arginine is not symmetric. In this work, the asymmetry of the N^∊^—C^ζ^—N^η1^ and N^∊^—C^ζ^—N^η2^ valence angles in the guanidinium moiety is confirmed. In addition, it was found that the C^δ^ atom can deviate significantly (more than 20°) from the guanidinium plane. This requires the relaxation of the planar restraint for the C^δ^ atom, as it otherwise causes the other atoms in the group to compensate by distorting the guanidinium core plane. A new set of restraints for the arginine side chain have therefore been formulated, and are available in the software package *Phenix*, that take into account the asymmetry of the group and the planar deviation of the C^δ^ atom. This is an example of the need to regularly revisit the geometric restraint libraries used in macromolecular refinement so that they reflect the best knowledge of the structural chemistry of their components available at the time.

## Abstract 2

The geometry of arginine shows more complexity than is accommodated by the standard restraints.

## 1. Introduction   

**Figure 1:**
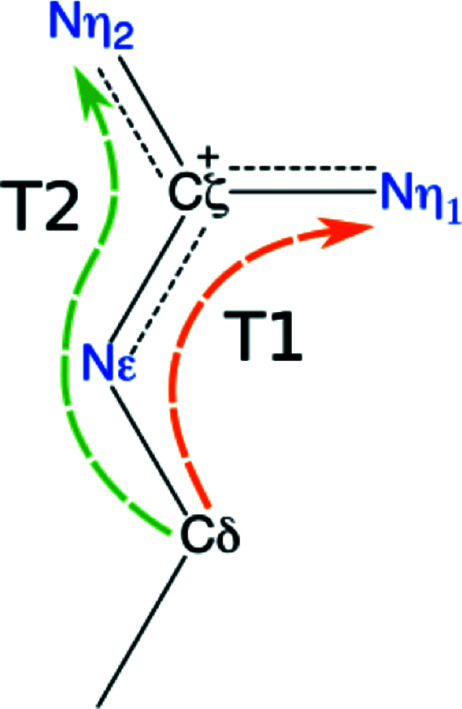
Diagram of the guanidinium moiety that terminates the side chain of arginine, including a schematic representation of the T1 and T2 torsion angles.

With 12 side-chain atoms and a molecular weight of about 174 Da, arginine is one of the largest standard amino acids. From a geometric viewpoint, arginine is very interesting because of the guanidinium group [–NH–C–(NH_2_)_2_^+^] in its side chain (Fig. 1 ▸). At physiological pH, guanidinium is always protonated and positively charged. Arginine is therefore often involved in salt bridges with negatively charged residues such as aspartic and glutamic acids. As the guanidinium group is hydrophilic, arginine residues are often located on the surface of the protein, so that the side chain can point towards solvent and form hydrogen bonds. Arginine is also very flexible: it has four χ angles (C^α^–C^β^, C^β^–C^γ^, C^γ^–C^δ^ and C^δ^–N^∊^) that yield 60 allowed rotameric configurations (Hintze *et al.*, 2016 ▸). This inherent flexibility and the fact that arginine is frequently located on the surface, where it is not sterically confined by neighbouring residues, often causes the density in crystallo­graphic Fourier maps to be partly or completely missing for many atoms of the side chain. As a consequence, arginine side chains can be difficult to model in crystallographic structures. As a result of the typically low observation-to-parameter ratio and the lack of high-resolution data in macromolecular crystallography, stereochemical restraints are required to maintain the correct geometry of arginine residues during crystallo­graphic refinement.

Refinement, which is driven by both restraints and experimental data, should result in a chemically reasonable structure. All refinement programs use geometry restraints, which provide *a priori* stereochemical information about the structural units of macromolecules. At high resolution (better than 1 Å), the experimental data typically provide sufficient information to produce accurate atomic coordinates (with the exception of flexible and disordered regions). However, at lower resolution (worse than 2.5–3 Å) geometry restraints are especially important because they dominate over the sparse experimental data. Therefore, geometry restraints need to be chemically accurate and their uncertainty, as indicated by the estimated standard deviations (e.s.d.s), should be sufficient to allow the experimental data to guide the refinement to a chemically reasonable result.

It is common practice to monitor the root-mean-squared deviations (r.m.s.d.s) from the geometry-restraint targets used in refinement to ensure that the weighting between experimental data and geometric information is reasonable. At low resolution, the r.m.s.d. values are typically small (approaching zero) as there are insufficient data to determine deviations from ideal geometry. At high resolution, the r.m.s.d. values can be larger when there is sufficient experimental data to define geometries that truly deviate from the library targets. A related metric for assessing the results of refinement is the r.m.s.*Z* (*Z*-score), which is an r.m.s.d. value that is normalized by the standard deviation of the restraint from the library. It is a dimensionless value that ideally should range from near zero for low-resolution models to approaching 1.0 for an ordered model based on high-resolution data.

Some geometry restraints have close chemical equivalents, such as bond lengths and valence angles. In contrast, the planarity restraint is less directly related to chemistry. It is commonly used to enforce planar structures arising from *sp* ^2^ hybridization. The π-orbital electrons of an *sp* ^2^-hybridized atom are repulsed by the bonded atoms, resulting in a planar structure. Naïvely, this planarity could be maintained by having the sum of the angle ideal values around the central atom summing to 360°. In practice, this approach fails to enforce planar geometry because each angle is implemented as a statistically independent quantity. This is remedied by adding a harmonic coplanarity restraint defined by reference to the best plane through the atomic positions within the scope of that restraint. The ‘ideal’ position of each atom is in the plane.

Engh & Huber (1991 ▸) generated ideal values for bonds and valence angles in standard amino acids to be used in macromolecular refinement with *X-PLOR* (Brünger, 1992 ▸). The values of the restraints have been updated (Engh & Huber, 2001 ▸), but the symmetry of the guanidinium moiety around the N^∊^—C^ζ^ bond was always enforced, *i.e.* to nearly identical values for the valence angles between the N^*i*^—C^ζ^—N^*j*^ atoms [N^∊^—C^ζ^—N^η1^, 120.3 (0.5)°; N^∊^—C^ζ^—N^η2^, 120.3 (0.5)°; N^η1^—C^ζ^—N^η2^, 119.4 (1.1)°].^1^ However, the guanidinium group in the arginine side chain is not expected to have this symmetric geometry, as the chemical environment of the group is not symmetric: the *cis* configuration leads to repulsion between the N^η1^ and C^δ^ atoms, causing a larger bond angle for N^∊^—C^ζ^—N^η1^. This asymmetry is well known. The *Handbook of Biochemistry and Molecular Biology* (Vijayan, 1976 ▸) reported the N^∊^—C^ζ^—N^η1^ and N^∊^—C^ζ^—N^η2^ valence angles as 121.5° and 119.3°, respectively. We also note that the libraries distributed with the *PROLSQ* (Hendrickson & Konnert, 1980 ▸) and *TNT* (Tronrud *et al.*, 1987 ▸; Tronrud, 1997 ▸) refinement packages contain asymmetric values that are indistinguishable from those given by Vijayan (1976 ▸). Their valence angles for the *cis* N atom (N^η1^) were therefore slightly larger than the ideal 120.3° proposed by Engh and Huber. However, current widely used macromolecular refinement packages, such as *Phenix* (Liebschner *et al.*, 2019 ▸) and *REFMAC* (Murshudov *et al.*, 2011 ▸), use the Engh and Huber restraints or derivations of them (Vagin *et al.*, 2004 ▸). Therefore, they all restrain the guanidinium group with symmetric valence angles.

Recently, Malinska *et al.* (2016 ▸) revisited the geometry of the guanidinium group in arginine. By analyzing high-resolution entries in the Protein Data Bank (PDB; wwPDB Consortium, 2019 ▸), they reported that the moiety is not symmetric. These results from the PDB analysis were corroborated by a search of the Cambridge Structural Database of small molecules.

Guanidine is a planar molecule resulting from a resonance structure of the three C—N bonds. This planarity extends to the H atoms, and once the moiety is bonded to the amino acid the effect of the π-electrons also extends to the C^δ^ atom. As a consequence, in addition to bonds and valence angles from Engh and Huber, the refinement restraints contain a planarity restraint for the three N atoms, two C atoms and five H atoms in the guanidinium moiety. The group has also a torsion-angle restraint involving the C^δ^—N^∊^—C^ζ^—N^η1^ atoms. In particular, the planar restraint includes the C^δ^ atom that bonds to N^∊^, thus replacing an H atom of guanidine. We note that in contrast to the asymmetry of the valence angles involving C^ζ^, no refinement package has deviated from this notion of uniform planarity for the guanidinium moiety.

However, instances of nonplanar guanidinium groups in arginine can be found in the PDB. A search of the Protein Geometry Database (Berkholz *et al.*, 2010 ▸) for arginine residues in models with better than 1.2 Å resolution revealed instances where the C^δ^ atom deviates more than 20° from the guanidinium plane. The nonplanarity of the arginine side chains is supported by the electron density.

**Figure 2:**
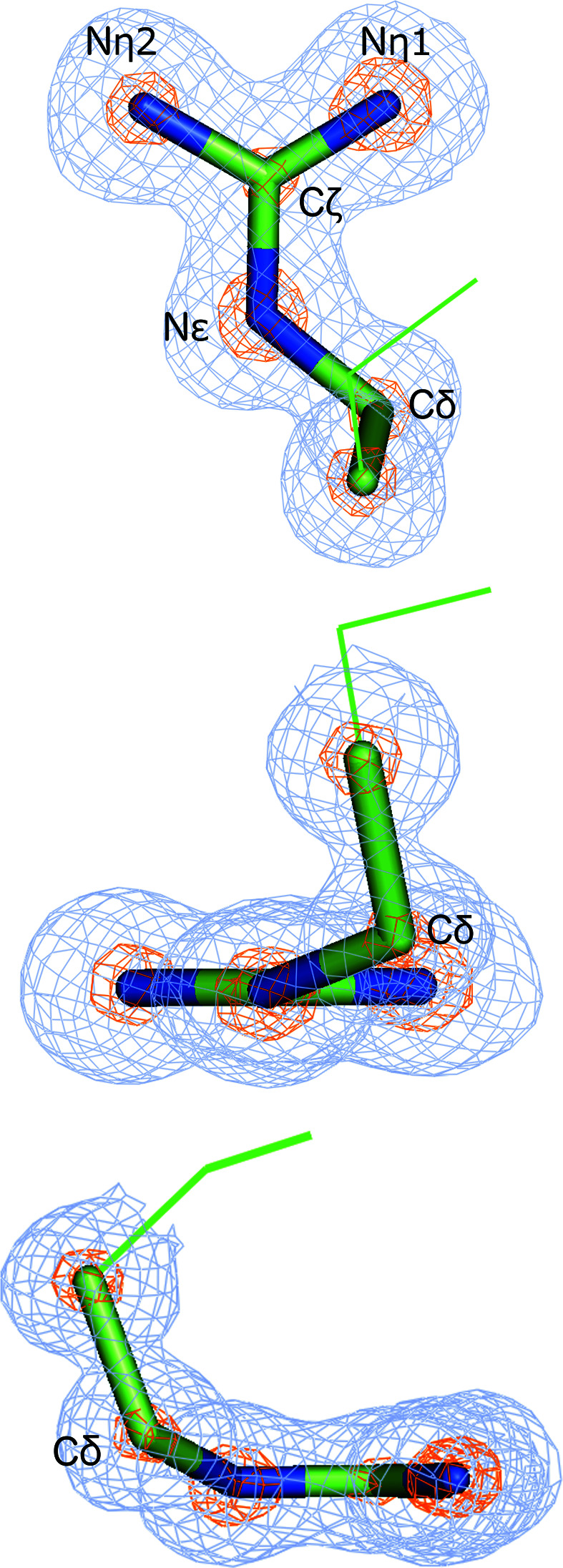
The C^δ^ atom can deviate significantly from the guanidinium plane in arginine. Three views of arginine (Arg242) in PDB entry 2xfr (0.97 Å resolution). Light blue, 2*mF*_obs_ − *DF*_model_ map at 1 r.m.s. contour. Orange, 2*mF*_obs_ − *DF*_model_ map at 5 r.m.s. contour. The locations of the C^α^ and C^β^ atoms are shown with lines.

One example of such a deviation is PDB entry 2xfr (Rejzek *et al.*, 2011 ▸), which was determined at 0.97 Å resolution. The C^δ^ atom in Arg242 deviates from planarity by approximately 22°, as measured by the C^δ^—N^∊^—C^ζ^—N^η1^ torsion angle. The atomic positions and therefore the distortion of the plane are clearly justified by the 2*mF*_obs_ − *DF*_model_ Fourier map (Fig. 2 ▸). Notably, the other atoms in the guanidine group remain visibly planar, indicating that the C^δ^ atom is more flexible. The residue is otherwise not an outlier, as it is in the favoured region of rotamer conformations and has no clashes.

The examples of planar deviations found in the Protein Geometry Database suggested that the planar restraint in guanidine needs modification in order to account for flexibility. Therefore, we analysed small-molecule compounds in the Cambridge Structural Database (CSD; Groom *et al.*, 2016 ▸) and performed systematic refinements of macromolecular structures in the PDB to quantify the flexibility of the C^δ^ atom in the guanidine group and to create a revised set of restraints.

## 2. Methods   

To obtain reliable small-molecule geometries, the CSD was searched for the guanidinium moiety (Fig. 1 ▸) and the resulting geometries (bond lengths, valence angles and torsion angles) were analysed. The CSD search and the geometry analysis were performed using the programs *Conquest* and *Mercury* (Bruno *et al.*, 2002 ▸, 2004 ▸) from the Cambridge Crystallographic Data Centre software suite. The search can be repeated using the script and settings provided in Supplementary Figs. S1 and S2. The analysis revealed that in small molecules the C^δ^ atom can deviate significantly from the plane imposed on the guanidinium moiety by the geometry restraints for proteins (see Section 3). This led us to formulate a new set of restraints for the guanidine group in arginine and to test the new restraints in macromolecular refinement.

To test the new arginine restraints, we refined models from the PDB with two different sets of restraints. The first set (‘standard’) uses the restraints for arginine from the Monomer Library, which is the standard restraints library for the refinement of macromolecules in *Phenix*. Here, the guanidinium group is restrained to be symmetrical and planar (Table 1 ▸). The second set of restraints (‘flexible’) includes asymmetric valence angles for the guanidinium group and allows the C^δ^ atom to deviate from the plane by using a larger standard deviation (0.095 Å instead of 0.020 Å). This corresponds to a C^δ^—N^∊^—C^ζ^—N^η1^ torsion (equivalent to a deviation from the guanidinium plane) of approximately 5°. All refinements were performed using *phenix.refine* (Afonine *et al.*, 2012 ▸). Coordinate and experimental data files were obtained from the PDB that met the following criteria: resolution better than 3.05 Å, data completeness >90%, data are not twinned, *R*_work_ < 30%, *R*_free_ < 35% and *R*_free_ − *R*_work_ > 1.5%. For entries with resolutions of better than 1.05 Å, the *R*_free_ − *R*_work_ criterion was changed to >0.5%. By using these criteria, we excluded suspicious entries and low-resolution data, allowing automatic refinement strategies with default options. H atoms were added to the models using *Phenix ReadySet!*. Ligand restraints were generated by *Phenix eLBOW* (Moriarty *et al.*, 2009 ▸).

Each model was then subjected to ten macrocyles of refinement using the default strategy in *phenix.refine* for the refinement of coordinates, atomic displacement parameters (ADP) and occupancies. Nondefault refinement options included optimization of the weight between the experimental data and the geometry restraints. In addition, anisotropic ADPs were used for non-H protein atoms at resolutions better than 1.55 Å and for water O atoms at resolutions better than 1.25 Å. The quality of the resulting models was assessed numerically using *MolProbity* (Williams *et al.*, 2018 ▸) in *Phenix*. To filter out problematic structures, refined models with a clashscore of greater than 12 were not included in the analysis. The results were grouped into resolution bins of width 0.1 Å. Resolution bins with less than 30 refined structures were not taken into account. This led to a total of 26 557 protein structures refined with conventional and modified arginine restraints.

## 3. Results and discussion   

### 3.1. Search for guanidinium in the CSD   

The search of the CSD for guanidinium (Fig. 1 ▸) resulted in 153 entries with 204 instances of the moiety. Some instances had geometric parameters that deviated significantly from the average. Visual inspection of these entries often revealed erroneous results (for example, the protonation state in the entry was not consistent with that in the search molecule; see, for example, entries CESPAR and COXYET) or an unusual chemical environment (for example, a sulfate coordinated to the guanidinium; see, for example, entries QAFTUN and SUXYUF).

As the process of manually examining the extrema and removing unreliable entries from the result list is not tractable, a statistically robust outlier-rejection method using the interquartile range, Tukey’s fences (Beyer, 1981 ▸), was applied to the torsion angles C^δ^—N^∊^—C^ζ^—N^η1^ (T1), C^δ^—N^∊^—C^ζ^—N^η2^ (T2) and T1 − T2 (which are the focus of this analysis; Fig. 1 ▸). This approach reduced the number of entries to 140 and the number of instances to 180. We note that the automatic process removed the entries already listed above and several more with similar issues. The removal of outliers discarded the entries with the most nonplanar guanidinium groups. In other words, it led to the removal of examples that support the flexibility of the moiety. We note that two instances of removed entries, caused by the presence of a sulfate ion causing distortion (QAFTUN and SUXYUF), are not outside the realm of possibility in proteins. Therefore, the outlier-removal process makes the set of entries more planar and, therefore, less extreme.

The values for bond lengths, valence angles and torsion angles for guanidine moieties in small molecules are summarized in Table 1 ▸. Histograms of the internal coordinate values are given in Supplementary Figs. S3–S5. The average bond lengths are essentially identical to the values reported by Malinska *et al.* (2016 ▸). The valence angles differ by an insignificant amount, possibly owing to the addition of models to the CSD since the study was performed and to the different outlier-rejection procedures. The guanidinium valence angles are asymmetric, with values of 121.5° and 119.2° for N^∊^—C^ζ^—N^η1^ and N^∊^—C^ζ^—N^η2^, respectively.

To analyse the planarity of the guanidinium group, we examined the torsion angles T1 (C^δ^—N^∊^—C^ζ^—N^η1^) and T2 (C^δ^—N^∊^—C^ζ^—N^η2^). The difference between T1 and T2 measures the planarity of the core moiety (N^∊^, C^ζ^, N^η1^ and N^η2^). The average of T1 − T2 in CSD guanidinium structures is 180.0 (1.2)°, meaning that the core moiety is indeed planar, with no entry deviating more than 3.2° from the plane. On the other hand, the torsion angles T1 and T2 have a standard deviation of 6.6° each, with a maximum absolute deviation of 16.2° and 16.7° for T1 and T2, respectively. This flexibility clearly shows that the C^δ^ atom has a propensity to deviate from the plane of the core moiety.

### 3.2. New features added to *Phenix*   

#### 3.2.1. New arginine restraints   

A feature of the implementation of restraints in *Phenix* makes it possible to easily add a flexible guanidinium planar restraint: *Phenix* allows the planar restraint to have a different estimated standard deviation (e.s.d.) value for each atom in a plane. The e.s.d. value for all atoms in the guanidinium plane is 0.02 Å for the standard restraints. In the case of the flexible restraints, an e.s.d. of 0.095 Å for the C^δ^ atom allows it to bend out of the plane to approximate the flexibility found in the molecules in the CSD (approximately 5°). The implied e.s.d. of the torsion angle of about 5° ensures that large deviations, as seen in the example described in Section 1 (PDB entry 2xfr), are allowed if supported by experimental data. Along with the relaxed planarity e.s.d. for the C^δ^ atom, the bond and angle values from the CSD analysis are used as a new set of restraints for the guanidine group of arginine (Table 1 ▸). Note that in both the original arginine restraints and the modified restraints, the T1 torsion angle is restrained to zero degrees with an e.s.d. of 10°. This is not a limiting restraint in either case.

#### 3.2.2. Consistent IUPAC atom naming for arginine   

One consequence of the asymmetry of the guanidinium group is that the N^η1^ and N^η2^ atoms need to be assigned the appropriate names. In compliance with the IUPAC convention for atom labelling, the N^η1^ atom should be always in *cis* configuration in comparison to the C^δ^ atom. Code was added to *Phenix* that automatically renames the N atoms (and the associated H atoms) if necessary. The parameter flip_symmetric_amino_acids controls the atom labelling in arginine and defaults to True from *Phenix* version dev-3951.

### 3.3. Refinement of macromolecules and comparison of standard arginine restraints versus flexible restraints   

To test whether a planar restraint with more flexibility for the C^δ^ atom is appropriate for arginine in proteins, we performed test refinements on structures deposited in the PDB. The 26 557 refined structures had experimental data resolutions between 0.85 and 3.05 Å (Fig. 2 ▸, inset).

The interpretation of the refinement results after applying different sets of restraints is a subtle matter. Global quality indicators such as *R* factors, clashscores or global bond and angle r.m.s.d. values are only marginally affected if the restraints to a single residue are changed. This is particularly true for arginine residues because they constitute only a small fraction of any given model. Instead, it is more appropriate to analyse bond, valence-angle, torsion-angle and planar r.m.s.d. and r.m.s.*Z* values for the modified restraints. While doing this, it is important to remember that r.m.s.d. and r.m.s.*Z* values should be also interpreted in the context of resolution. At low resolution, the model can be made to agree with any reasonable restraint without violating the fit to the blurry density; r.m.s.d. values are expected to be low even for restraints that do not completely respect the chemistry. At medium to high resolution (1.5–2.5 Å), restraints and data have a similar weight in refinement. The data may contain enough information to drive the model towards a chemically meaningful geometry, but if the restraints are chemically unreasonable the r.m.s.d./r.m.s.*Z* values may increase. At ultrahigh resolution (better than 1 Å) the data dominate over the restraints, resulting in a model that is chemically correct, with r.m.s.d./r.m.s.*Z* trending towards higher values. However, not all restraints need to deviate from ideality at high resolution. Indeed, if the r.m.s.d. value of a certain restraint remains low at high resolution then this ideal value is appropriate for the majority of models. Therefore, when comparing sets of restraints after refinement, it is appropriate to focus on the medium- to ultrahigh-resolution range, as the low-resolution range will generally have low r.m.s.d./r.m.s.*Z* values.

**Figure 3:**
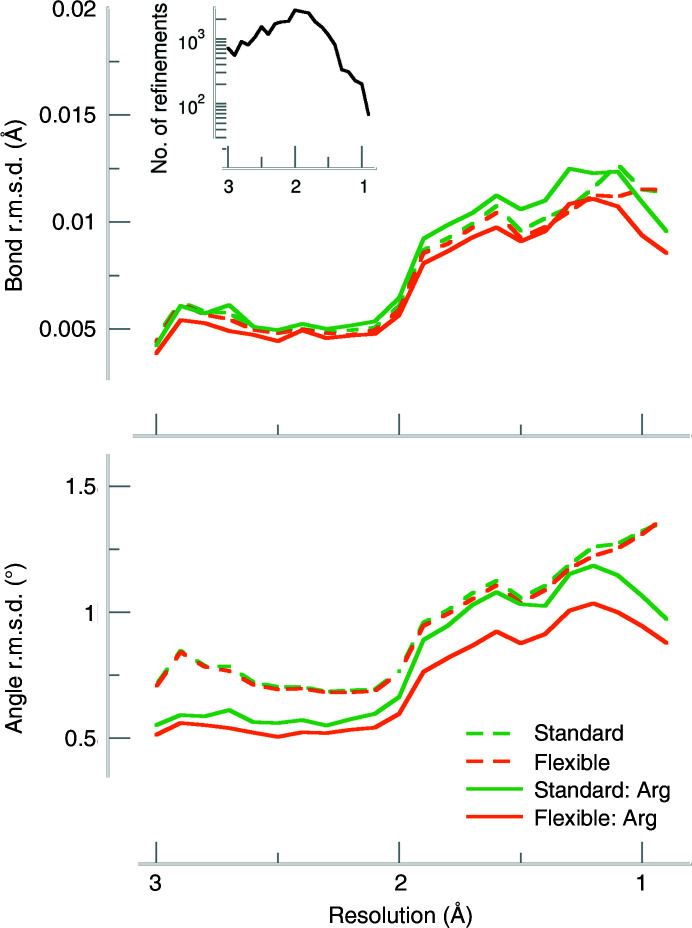
Bond-length and angle r.m.s.d. values averaged in 0.1 Å resolution bins. Refinements with standard arginine restraints are plotted using green lines and flexible restraints are shown in orange. The r.m.s.d. values for the whole model are shown as dashed lines, while the arginine-only r.m.s.d. values are shown as solid lines. The inset shows the number of refinements in each resolution bin.

If the restraint target values are modified while keeping the e.s.d. constant, the r.m.s.d. and r.m.s.*Z* values are a good indicator that reflects whether the new target values lead to a less strained model. This is what can be observed for the bond and angle restraints in the guanidinium group: the bond and angle r.m.s.d.s are lower for flexible restraints (*i.e.* modified bonds and valence angles) than for standard restraints in the resolution range 3.0 Å and better (Fig. 3 ▸).

One must investigate the effect of adding flexibility to a planar restraint in a different way, as only the e.s.d.s are changed, not the target values themselves. The e.s.d. of the C^δ^ atom was increased and as a consequence the atom can move more freely (*i.e.* out of plane) during refinement. This means that the r.m.s.d. in most cases increases as well. Therefore, instead of looking at the r.m.s.d./r.m.s.*Z* for a particular restraint (C^δ^) only, it is important to analyse the r.m.s.d./r.m.s.*Z* for the entire guanidinium group.

#### 3.3.1. C^δ^—N^∊^—C^ζ^—N^η1^ torsion angle   

**Figure 4:**
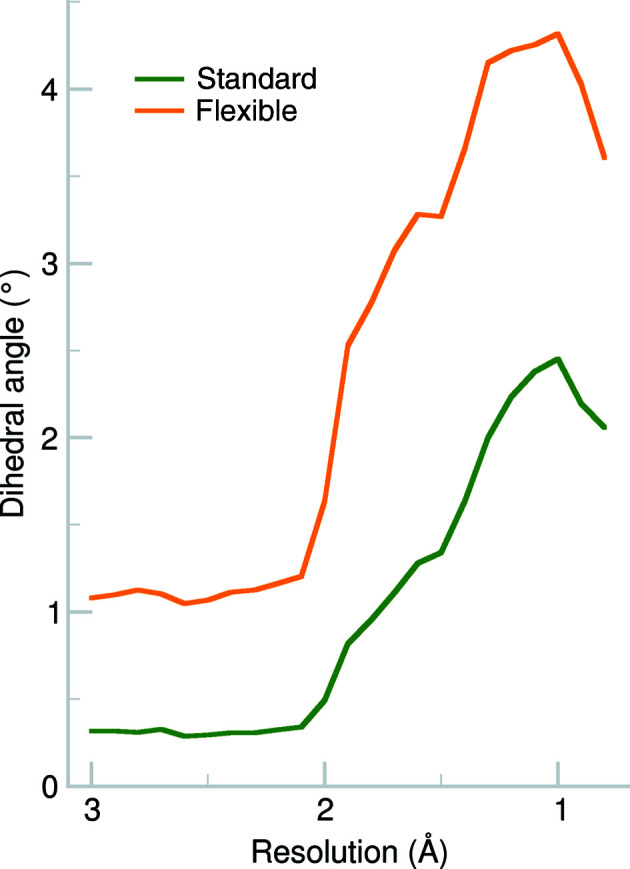
Values of the C^δ^—N^∊^—C^ζ^—N^η1^ torsion angle for the standard restraints (green) and flexible restraints (orange) in 0.1 Å resolution bins.

Fig. 4 ▸ shows the absolute deviation from zero of the C^δ^—N^∊^—C^ζ^—N^η1^ (T1) torsion angle in resolution bins. We note that even for the standard restraint, where the planar restraint is uniform across the plane, the T1 torsion angle has a mean of approximately 0.25° at resolutions worse than 2 Å, with a maximum of nearly 2.5° at high resolution. The flexible restraint results in a torsion-angle deviation of 1° in the medium-resolution range and of greater than 4° at high resolution. The torsion angle is therefore systematically larger when flexible arginine restraints were used. This behaviour is expected. The larger e.s.d. allows the atoms to deviate from the plane, with the deviation being more pronounced at higher resolution.

#### 3.3.2. C^δ^ deviations   

**Figure 5:**
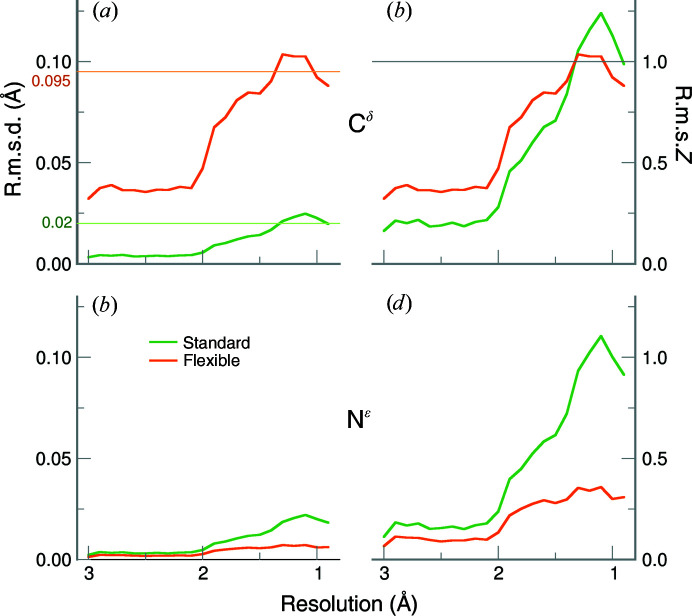
Planar r.m.s.d. (*a*, *c*) and r.m.s.*Z* (*b*, *d*) values for C^δ^ and N^∊^ atoms in the guanidinium moiety averaged in 0.1 Å resolution bins. Refinements using the standard restraints are shown as green lines, while orange lines denote the flexible restraints. Atom C^δ^ is shown in (*a*) and (*b*) and atom N^∊^ is shown in (*c*) and (*d*). The e.s.d. for C^δ^ is shown for the standard (0.020 Å) and flexible (0.095 Å) restraints for reference. The lower row is on the same scale as the upper graphs.

Fig. 5 ▸(*a*) shows the r.m.s.d. values of the planar restraint for the C^δ^ atom that reflect its deviation from the plane. (Supplementary Fig. S6 includes the standard error of the mean for all results in Fig. 5 ▸.) The r.m.s.d. is relatively close to zero at low resolution for the standard restraints, but increases up to 0.025 Å at resolutions better than 2 Å. The r.m.s.d. values for the flexible restraints are numerically larger, which is in line with the fact that the C^δ^ atom can now move more freely. As for the r.m.s.*Z* values of the C^δ^ planar restraint (Fig. 5 ▸ *b*), the C^δ^ atom deviates approximately one sigma value from the mean for both sets of restraints. We note that the standard restraint values have an r.m.s.*Z* of greater than 1.2 at high resolution, which is greater than for the flexible restraints, suggesting that the original restraint is too restrictive.

#### 3.3.3. N^∊^ deviations   

The N^∊^ atom r.m.s.d. and r.m.s.*Z* values show the opposite trend compared with the C^δ^ atom. The r.m.s.d. values for the N^∊^ atom (Fig. 5 ▸ *c*) are similar (close to zero) for both sets of restraints at resolutions worse than 2 Å. At resolutions better than 2 Å, the N^∊^-atom r.m.s.d. values are systematically larger for the standard restraints than for the flexible restraints. This indicates that the N^∊^ atom tends to be closer to the plane when the flexible restraints are used. Not surprisingly, there is a similar reduction in r.m.s.*Z* values for the N^∊^ atom (Fig. 5 ▸ *d*). The r.m.s.*Z* values are systematically smaller for the flexible restraints in all resolution ranges, with a significant reduction at a resolution of 2 Å and better. The drop in N^∊^-atom r.m.s.d. and r.m.s.*Z* values therefore suggests that the core moiety (N^∊^—C^ζ^—N^η1^—N^η2^) becomes more planar with the flexible restraints.

#### 3.3.4. N^η1^ and N^η1^ deviations   

**Figure 6:**
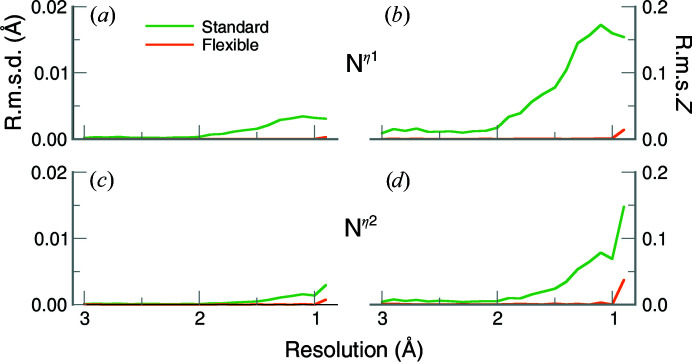
Planar r.m.s.d. (*a*, *c*) and r.m.s.*Z* (*b*, *d*) values for N^η1^ and N^η2^ atoms in the guanidinium moiety averaged in 0.1 Å resolution bins. Refinements using the standard restraints are shown as green lines, while orange lines denote the flexible restraints. Atom N^η1^ is shown in (*a*) and (*b*) and atom N^η2^ is shown in (*c*) and (*d*). The lower row is on the same scale as the upper graphs.

Investigating the N^η1^ and N^η2^ atoms of the guanidinium moiety provides additional insights (Fig. 6 ▸ and Supplementary Fig. S7). The r.m.s.d.s are essentially zero at resolutions worse than 2 Å. At resolutions better than 1.5 Å, the standard-restraint r.m.s.d. values increase slightly (approaching 0.003 Å) for the N^η1^ and N^η2^ atoms. Although this is remarkably small, it is still larger than the r.m.s.d. values using the flexible restraints, which are essentially zero over the entire resolution range. The r.m.s.*Z* values show the same trend: the values for the flexible restraints are systematically smaller. Therefore, as the N^η1^ and N^η2^ atoms deviate only marginally from the guanidinium plane, this further suggests that the core moiety is flat.

The behaviour of the C^δ^, N^∊^, N^η1^ and N^η2^ atoms can be summarized as follows. For the standard restraints, the non-C^δ^ atoms (N^∊^, N^η1^ and N^η2^) compensate for the lack of freedom of movement of the C^δ^ atom by deviating ever so slightly from the guanidinium plane. The new flexible restraints allow the C^δ^ atom to move away from the plane, while the other atoms can relax into the planar core moiety.

#### 3.3.5. High-resolution example   

For the Arg242 residue in PDB entry 2xfr, refinement with flexible arginine restraints increases the T1 torsion angle from 19° to 24° using the standard and flexible restraints, respectively, while increasing the chemically meaningful planarity of the core group, where the deviation of the N^∊^ atom is reduced from 0.13 to 0.02 Å.

## 4. Conclusions   

Our analysis of small molecules in the CSD reiterates that the guanidinium moiety is asymmetric (N^∊^—C^ζ^—N^η1^, 121.5°; N^∊^—C^ζ^—N^η2^, 119.2°). Importantly, this analysis also revealed that the C^δ^ atom deviates from the plane of the guanidinium group. This plane is typically enforced in crystallographic refinement as a geometry restraint. Based on the bond lengths and valence angles from the CSD, as well as on the propensity of the C^δ^ atom to deviate from the plane, we formulated a revised set of geometry restraints for the guanidinium group in arginine. To test the impact of these new restraints, we performed refinements of 26 557 PDB entries against X-ray data in the resolution range 0.85–3.55 Å. Arginine bond and angle r.m.s.d.s improve with the new sets of restraints. The C^δ^ atom, which is allowed to deviate more from the plane with an increased e.s.d., indeed has a propensity to move further away from it. However, this increased flexibility of the C^δ^ atom simultaneously allows the guanidinium core group to become more planar. While the new set of restraints will generally not affect global quality indicators of refinement, it will lead to more chemically meaningful models. We note that the increased flexibility of the arginine side chain can affect the interpretation of hydrogen-bond networks, which are often important for catalytic mechanisms. We therefore suggest that arginine restraints should be updated broadly in refinement and validation programs. The new set of restraints is available in *Phenix* version dev-3951 and later.

## Supplementary Material

Supplementary Figures. DOI: 10.1107/S2059798320013534/qj5005sup1.pdf

## Figures and Tables

**Table 1:** Bond lengths (Å), valence angles (°) and torsion angles (°) with standard uncertainties in the guanidine groups in arginine from various sources Two sets of geometric values are from Malinska *et al.* (2016 ▸).

	C^δ^—N^∊^	N^∊^—C^ζ^	C^ζ^—N^η1^	C^ζ^—N^η2^	C^δ^—N^∊^—C^ζ^	N^∊^—C^ζ^—N^η1^	N^∊^—C^ζ^—N^η2^	N^η1^—C^ζ^—N^η2^	C^δ^—N^∊^—C^ζ^—N^η1^ (T1)	C^δ^—N^∊^—C^ζ^—N^η2^ (T2)	T1 − T2
This work: CSD 2019											
Mean	1.458	1.326	1.323	1.330	124.4	121.5	119.2	119.3	0.9	180.7	180.0
R.m.s.d.	0.011	0.011	0.010	0.009	1.4	0.9	0.9	0.9	6.7	6.7	1.2
Min	1.421	1.292	1.302	1.300	118.9	119.0	116.4	117.2	−16.2	163.3	176.9
Max	1.557	1.361	1.370	1.375	127.0	123.7	122.5	122.5	13.3	194.4	183.0
Malinska: PDB survey											
Mean	1.458	1.327	1.325	1.328	124.9	121.3	119.2	119.6			
R.m.s.d.	0.012	0.011	0.013	0.012	1.4	1.0	1.0	1.0†			
Min	1.390	1.267	1.266	1.294	119.4	118.2	114.2	113.6			
Max	1.520	1.384	1.386	1.394	130.2	124.6	123.0	126.1			
Malinska: CSD 2016											
Mean	1.456	1.326	1.323	1.329	124.4	121.5	119.2	119.4			
R.m.s.d.	0.014	0.011	0.014	0.013	1.4	1.0	0.9	1.3			
GeoStd											
Ideal	1.460	1.329	1.326	1.326	124.2	120.0	120.0	119.7			
E.s.d.	0.018	0.014	0.018	0.018	1.5	1.9	1.9	1.8			

† Appears to be a typographical error in Malinska *et al.* (2016 ▸).

## Footnotes

1 The choice of having a symmetric guanidinium group possibly originates from the heritage of the use of the library use in molecular-dynamics (MD)-based refinement. With the large motions expected in an MD simulation, atom labels would either have to be regularly swapped, or a more complex force field would be required that would recognize the dependence of these valence angles on the associated torsion angle. Assigning symmetric valence angles is likely to have avoided this complication. Unfortunately, when the Engh and Huber library was used in other programs, the target values were not updated.
