# Supplementary material for: Arginine off-kilter: guanidinium is not as planar as restraints denote
Source: Acta Crystallogr D Struct Biol. 2020 Nov 24;76(Pt 12):1159–66. doi: 10.1107/S2059798320013534 (PMC7709202; doi:10.1107/S2059798320013534)
Supplement: Supplementary file 1 [file d-76-01159-sup1.pdf]

STRUCTURAL BIOLOGY

Volume 76 (2020)

Supporting information for article:

Arginine off-kilter: guanidinium is not as planar as restraints denote

Nigel W. Moriarty, Dorothee Liebschner, Dale E. Tronrud and Paul D. Adams

S1. CSD search details

Fig. S1 contains the .con file used to search the CSD. The addition filters are shown in Fig. S2.

S2. CSD results

Results of the CSD structure search for internal coordinates summarised in table 1 are displayed in Fig. S3-S5.

S3. Plane *rmsd* for atoms in guanidinium moiety

Standard error of the mean (SEM) error bars for the plots in Fig. 5 and 6 are shown in Fig. S6 and S7, respectively.

```
T1 *CONN
NFRAG -99
ELDEF CC = AA - D
AT1 C 3  T3        :XY 248 238
AT2 N 2 1         :XY 166 240
AT3 N 1 2         :XY 303 178
AT4 N 1 2         :XY 298 289
AT5 C 2 2 T4        :XY 122 297
AT6 CC 1         :XY 62 297
BO 1 3 99
BO 5 6 1
BO 2 5 1
BO 1 4 99
BO 1 2 99
GEOM
DEFINE V1 4 1 3
DEFINE V2 2 1 3
DEFINE V3 2 1 4
DEFINE V4 5 2 1
DEFINE T1 5 2 1 3
DEFINE T2 5 2 1 4
DEFINE B1 1 3
DEFINE B2 1 4
DEFINE B3 1 2
DEFINE B4 2 5
DEFINE T3 6 5 2 1
SYMCHK ON
ENANT NORMAL
END
```

Figure S1 Conquest .con file used to search the CSD database.

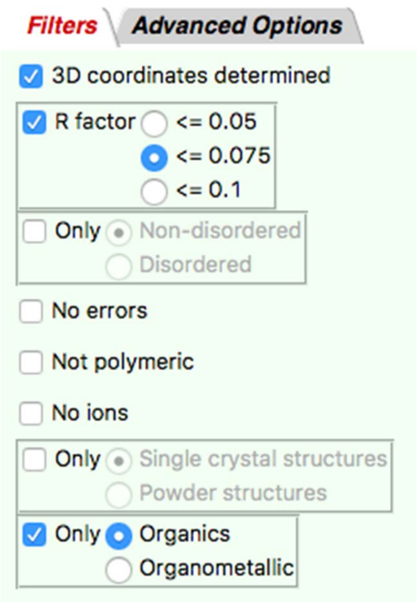

Figure S2 Search filters used for the CSD search.

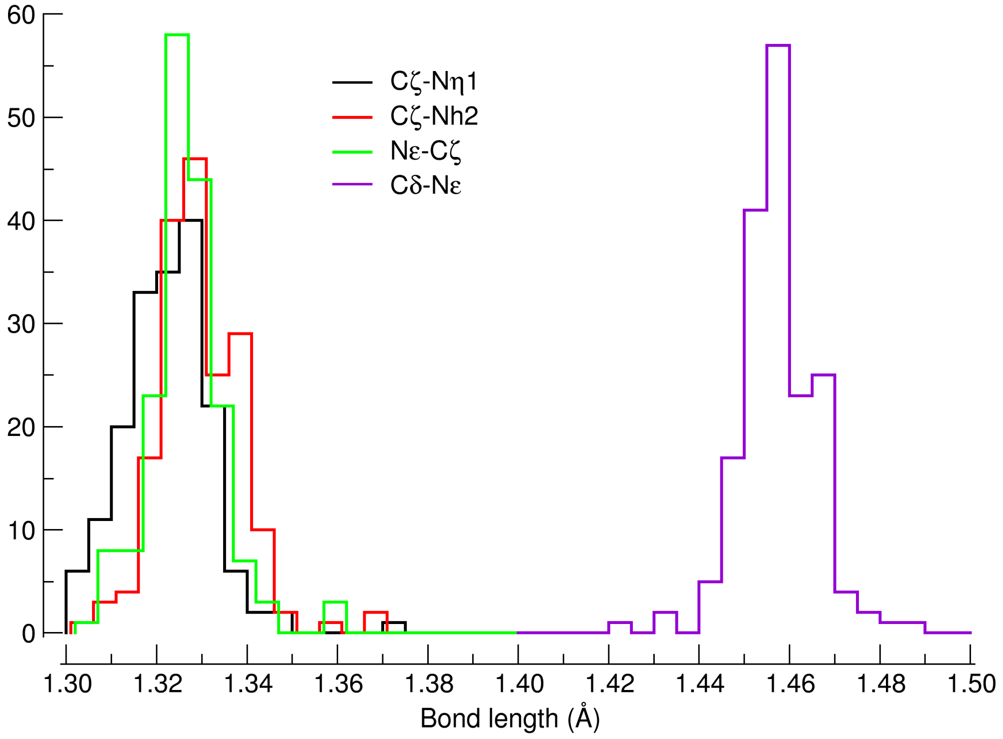

Figure S3 Bond lengths in the guanidinium group from the CSD search.

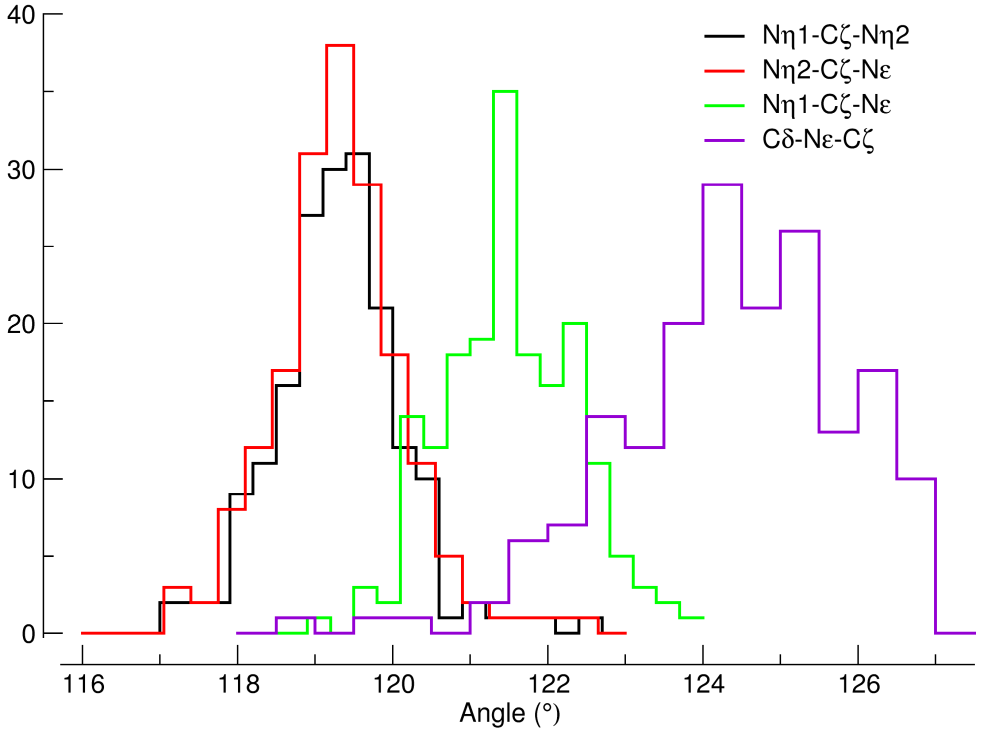

Figure S4 Valence angles in guanidinium from the CSD search.

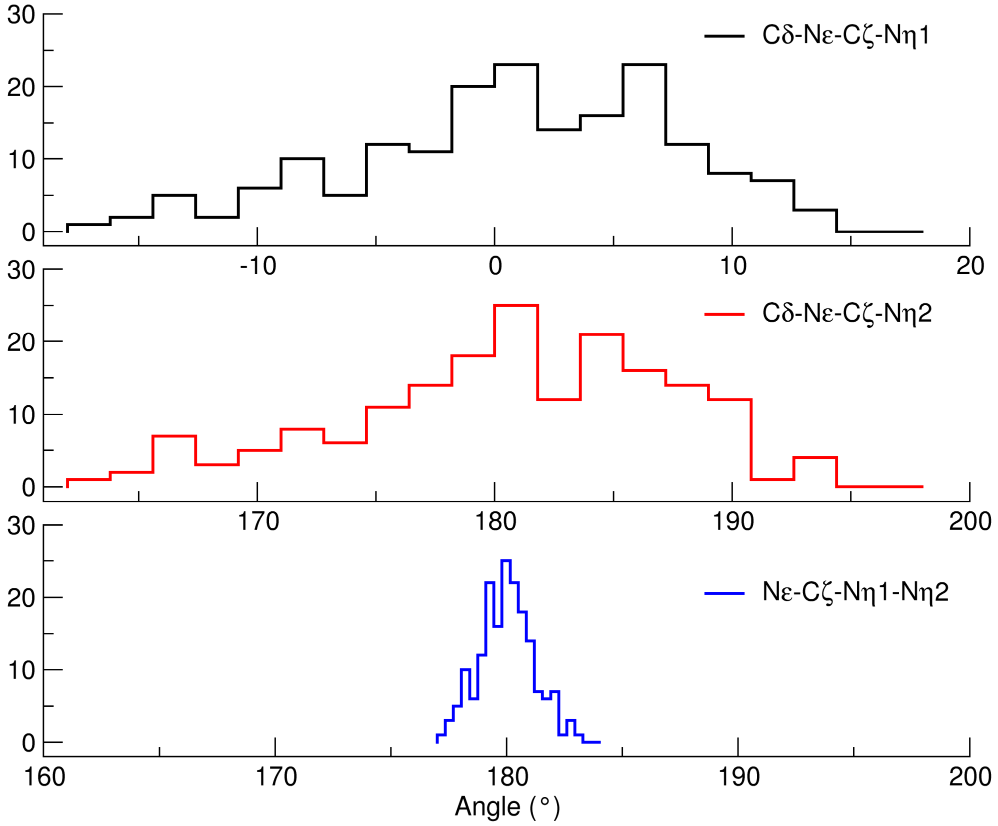

Figure S5 Dihedral angles in the guanidinium group from the CSD search.

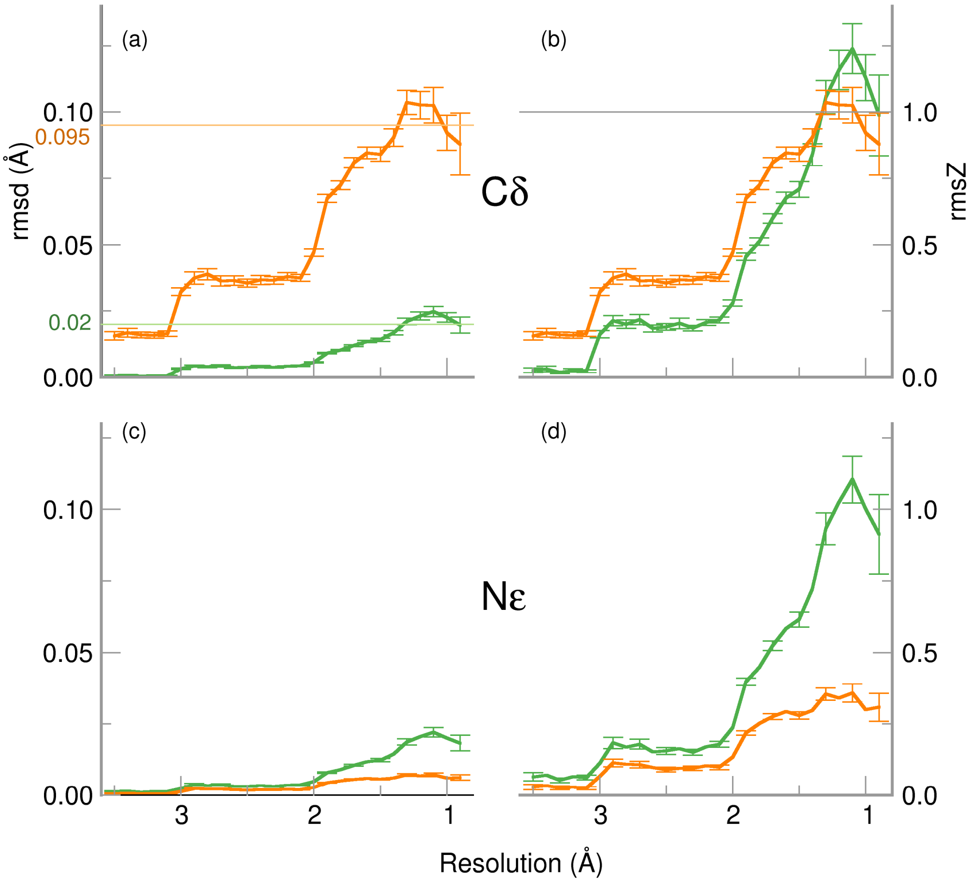

Figure S6 Standard Error of Mean for data points in Fig. 5.

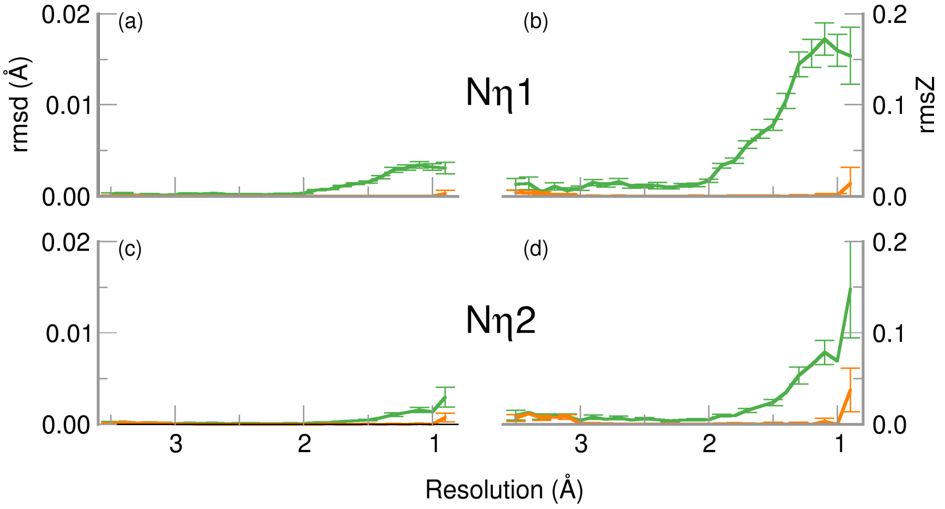

Figure S7 Standard Error of Mean for data points in Fig. 6.
